# Supplementary material for: Pyricularia’s Capability of Infecting Different Grasses in Two Regions of Mexico
Source: J Fungi (Basel). 2023 Oct 27;9(11):1055. doi: 10.3390/jof9111055 (PMC10672002; doi:10.3390/jof9111055)
Supplement: Supplementary file 1 [file jof-09-01055-s001.zip › Fig S1.pdf]

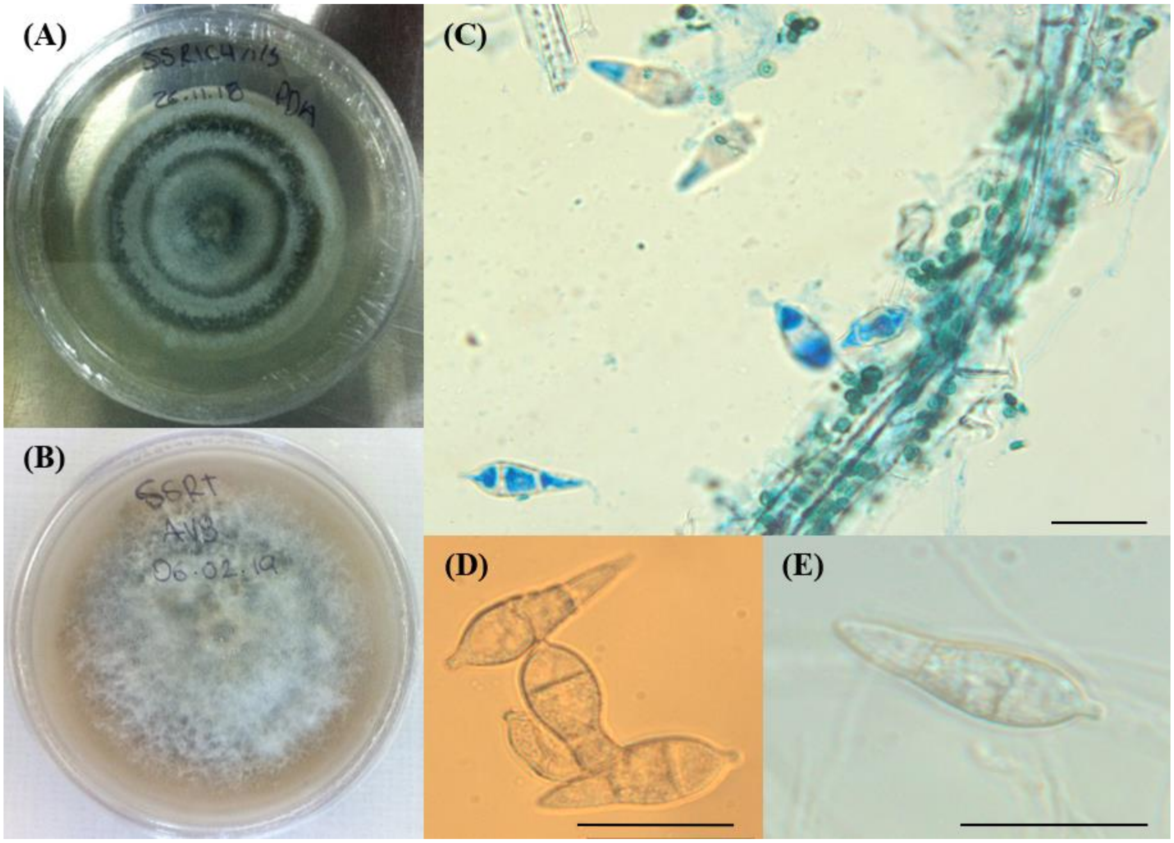

Figure S1. Representative morphologies of *Pyricularia* isolates by using the SR1 isolate. Growth on PDA agar showed a ringed sporulation pattern (**A**), and on V8 agar showed mycelia with black and white cottony masses (**B**). Different pictures (**C**, **D** and **E**) show the morphologic characteristics of the conidia associated whit this fungus: pyriform, narrowed toward the tip, rounded at the base, colorless, smooth with three septa, and a size average of 28.1 x 8.5 µm. Bars below indicate 20 µm.
