# Supplementary material for: Pyricularia’s Capability of Infecting Different Grasses in Two Regions of Mexico
Source: J Fungi (Basel). 2023 Oct 27;9(11):1055. doi: 10.3390/jof9111055 (PMC10672002; doi:10.3390/jof9111055)
Supplement: Supplementary file 1 [file jof-09-01055-s001.zip › Fig S2.pdf]

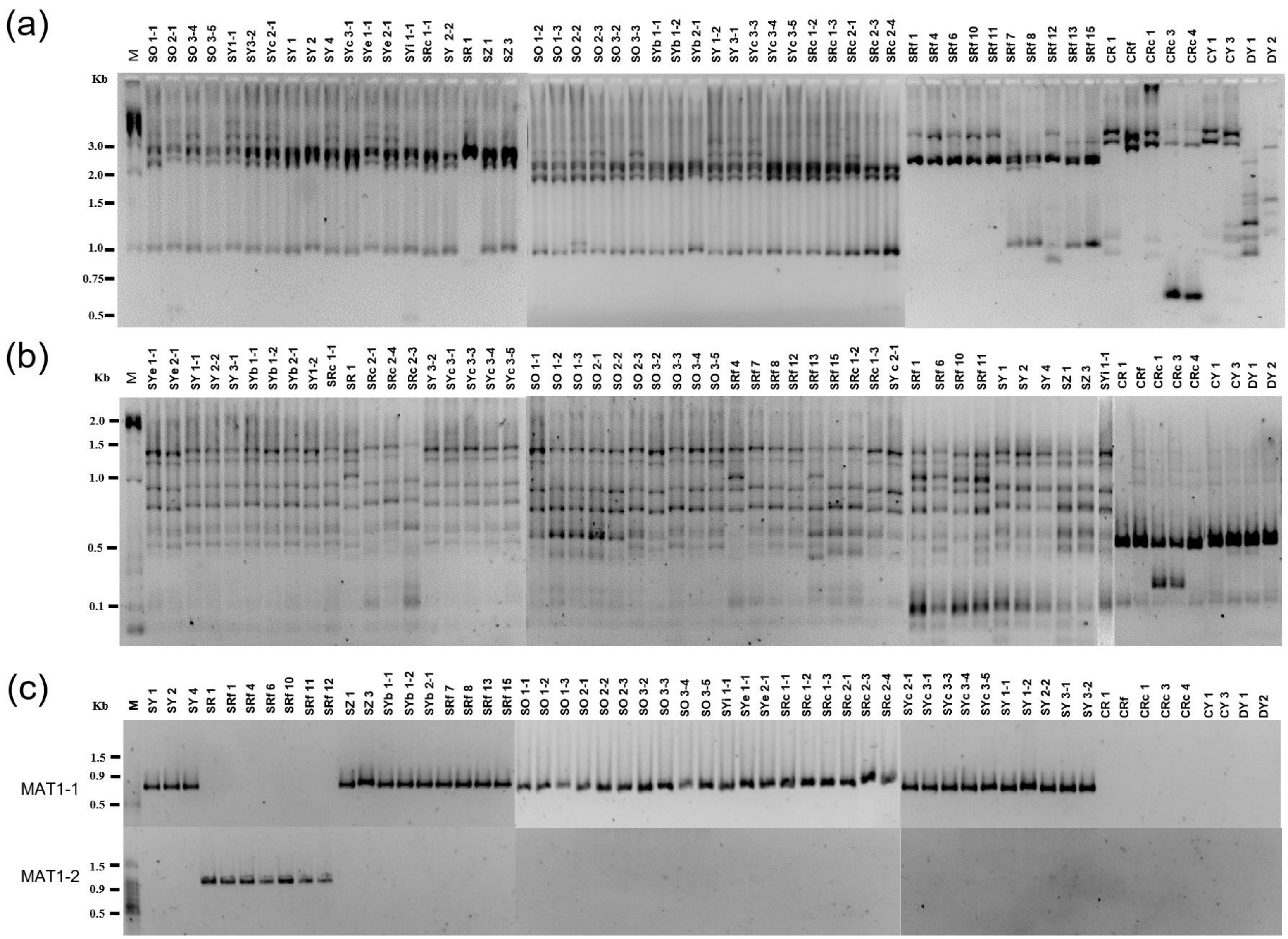

Figure S2. Genomic fingerprinting and mating type analysis. **A.** Pot2-TIR amplification profile showing band variability. M: Molecular marker ladder 1 kb (Promega, USA). **B**. ERIC-PCR amplification profiles showing band variability. M: Molecular marker ladder 1kb (Promega, USA). **C.** Mating type analysis for the *Pyricularia* isolates of this study. (Upper gel) *MAT1-1* (809bp), (Lower gel) *MAT1-2* (940pb). M: Molecular weight ladder (Promega100 bp), C: Negative control. All the gels were run at the same conditions.
