# Supplementary material for: Pyricularia’s Capability of Infecting Different Grasses in Two Regions of Mexico
Source: J Fungi (Basel). 2023 Oct 27;9(11):1055. doi: 10.3390/jof9111055 (PMC10672002; doi:10.3390/jof9111055)
Supplement: Supplementary file 1 [file jof-09-01055-s001.zip › Fig S3.pdf]

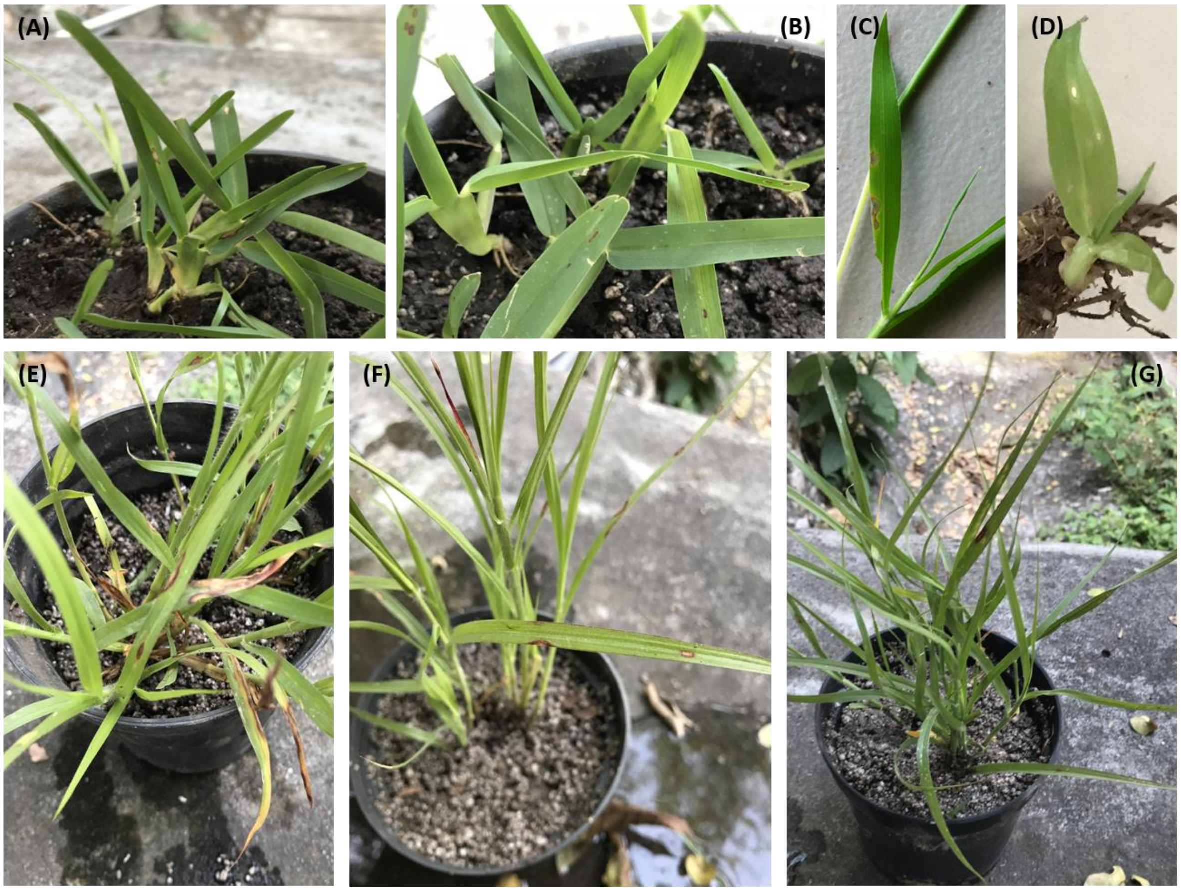

Figure S3. Representative images of the cross-infection assay by means of spore spraying in *S. secundatum*, *C. ciliaris* and *D. ciliaris* grasses. Lesions in leaves from *S. secundatum* grass produced by SY2 (**A**) and CR1 (**B**) isolates, respectively. Lesions in leaves from *D. ciliaris* grass produced by DY1 in mature (**C**) and young (**D**) leaves. Lesions in leaves from buffel grass (*C. ciliaris*) produced by CY1 (**E**), CR1 (**F**), and SR1 (**G**).
